# A *Moraxella* Virulence Factor Catalyzes an Essential Esterase Reaction of Biotin Biosynthesis

**DOI:** 10.3389/fmicb.2020.00148

**Published:** 2020-02-11

**Authors:** Qi Zeng, Qi Yang, Jia Jia, Hongkai Bi

**Affiliations:** Jiangsu Key Laboratory of Pathogen Biology, Department of Pathogen Biology, Nanjing Medical University, Nanjing, China

**Keywords:** *Moraxella catarrhalis*, methyl esterase, pimelate thioester, biotin synthetic pathway, bacterial virulence, BtsA

## Abstract

Pimeloyl-acyl carrier protein (ACP) methyl ester esterase catalyzes the last biosynthetic step of the pimelate moiety of biotin, a key intermediate in biotin biosynthesis. The paradigm pimeloyl-ACP methyl ester esterase is the BioH protein of *Escherichia coli* that hydrolyses the ester bond of pimeloyl-ACP methyl ester. Biotin synthesis in *E. coli* also requires the function of the malonyl-ACP methyltransferase gene (*bioC*) to employ a methylation strategy to allow elongation of a temporarily disguised malonate moiety to a pimelate moiety by the fatty acid synthetic enzymes. However, bioinformatics analyses of the extant bacterial genomes showed that *bioH* is absent in many *bioC*-containing bacteria. The genome of the Gram-negative bacterium, *Moraxella catarrhalis* lacks a gene encoding a homolog of any of the six known pimeloyl-ACP methyl ester esterase isozymes suggesting that this organism encodes a novel pimeloyl-ACP methyl ester esterase isoform. We report that this is the case. The gene encoding the new isoform, called *btsA*, was isolated by complementation of an *E. coli bioH* deletion strain. The requirement of BtsA for the biotin biosynthesis in *M. catarrhalis* was confirmed by a biotin auxotrophic phenotype caused by deletion of *btsA in vivo* and a reconstituted *in vitro* desthiobiotin synthesis system. Purified BtsA was shown to cleave the physiological substrate pimeloyl-ACP methyl ester to pimeloyl-ACP by use of a Ser117-His254-Asp287 catalytic triad. The lack of sequence alignment with other isozymes together with phylogenetic analyses revealed BtsA as a new class of pimeloyl-ACP methyl ester esterase. The involvement of BtsA in *M. catarrhalis* virulence was confirmed by the defect of bacterial invasion to lung epithelial cells and survival within macrophages in the Δ*btsA* strains. Identification of the new esterase gene *btsA* exclusive in *Moraxella* species that links biotin biosynthesis to bacterial virulence, can reveal a new valuable target for development of drugs against *M. catarrhalis*.

## Introduction

*Moraxella catarrhalis* is a Gram-negative, human-restricted opportunistic bacterial pathogen that colonizes the upper and lower respiratory tracts. *M. catarrhalis* can be carried asymptomatically (known as carriage), but can also causes otitis media in children and lower respiratory tract infections in adults with chronic obstructive pulmonary disease (Verduin et al., 2002). It is commonly found in a polymicrobial community with other pathogens such as *Streptococcus pneumoniae* and *Haemophilus influenzae*. The vast majority of clinical isolates of *M. catarrhalis* (>95%) are now resistant to the β-lactamase family of antibiotics that was once considered a front-line treatment for the disease (Masaki et al., 2011). Until now, an efficient vaccine against *M. catarrhalis* has not yet been developed.

**Figure 1:**
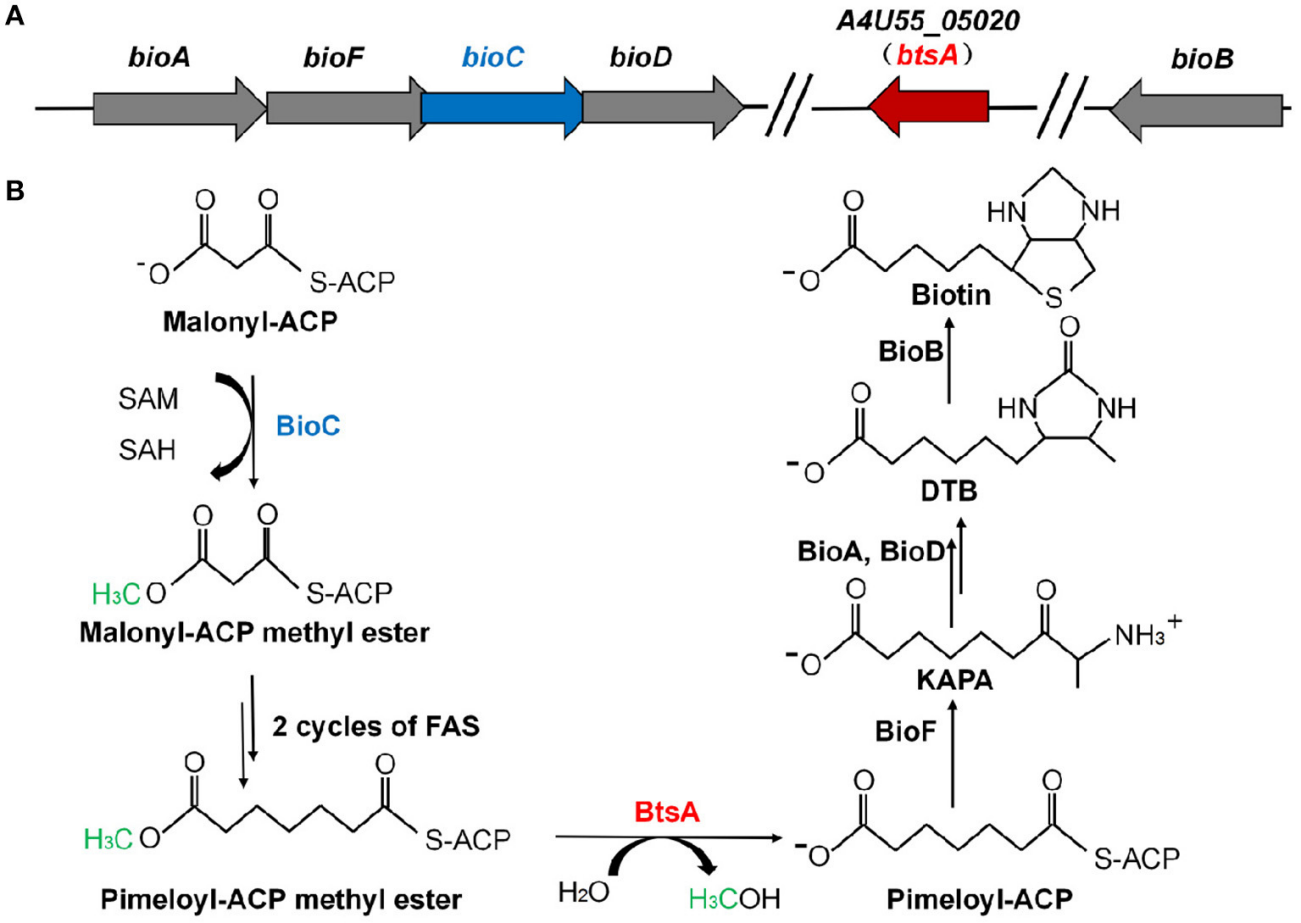
Genetic organization of biotin biosynthetic genes and the proposed model for the *M. catarrhalis* biotin biosynthetic pathway. **(A)** The operon of the biotin biosynthetic genes is shown. The *btsA* gene is colored red and the *bioC* gene is colored blue. **(B)** Scheme of the proposed *M. catarrhalis* biotin synthetic pathway. FAS denotes the fatty acid synthesis cycle.

Biotin (vitamin H or vitamin B7) is an essential micronutrient required in all living organisms (Beckett, 2007). It functions as a covalently-bound enzyme cofactor which mediates the transfer of CO_2_ during carboxylation, decarboxylation, and transcarboxylation reactions (Knowles, 1989; Attwood and Wallace, 2002). *In vivo*, biotin functions like a long “swinging arm” that transfers intermediates between two active sites of essential metabolic enzymes via covalent substrate channeling (Cronan, 2014). Biotin is synthesized *de novo* from the seven-carbon α,ω-dicarboxylate intermediate, pimelate, which is esterified with either CoA (pimeloyl-CoA) or acyl carrier protein (pimeloyl-ACP) (Lin et al., 2010; Cronan, 2014). Conversion of this common pimeloyl thioester precursor to biotin is carried out by four remarkably well-conserved enzymes (BioF, BioA, BioD, and BioB) (Figure 1B), that have been extensively worked out many years ago largely in *Escherichia coli* (Lin and Cronan, 2011; Cronan, 2014). In contrast to the late steps, the early steps responsible for synthesis of the pimelate moiety are quite diverse. The best clearly described synthetic pathway for the pimelate moiety is represented by the *E. coli* BioC-BioH pathway, which hijacks a fraction of the fatty acid biosynthetic capacity to make the pimelate moiety (Lin and Cronan, 2011; Cronan, 2018). BioC, a carboxyl-methyltransferase was found to initiate biotin synthesis by methylation of the free carboxyl group of a malonyl-ACP (Lin and Cronan, 2012). The methylated malonyl-ACP mimicks the substrate which is recognized by the enzymes of type II fatty acid biosynthesis (White et al., 2005) and is elongated for two cycles with addition of four carbon atoms to give a pimeloyl-ACP methyl ester. The promiscuous esterase BioH subsequently cleaves the methyl moiety to produce pimeloyl-ACP, which then enters the late steps of biotin synthetic pathway. Although BioH is considered as a “wild card” among biotin synthetic enzymes, it acts as a gatekeeper and blocks the further elongation of its physiological substrate (Agarwal et al., 2012). However, these enzymes that have BioH-like activity show marked sequence diversity among *bioC*-containing bacteria. Four other enzymes, BioG (Shi et al., 2016), BioK (Shapiro et al., 2012), BioJ (Feng et al., 2014), and BioV (Bi et al., 2016), have been discovered in bacteria that encode BioC but not BioH (Rodionov et al., 2002). All these esterases have been demonstrated to catalyze the cleavage of pimeloyl-ACP methyl ester *in vitro* and to rescue biotin synthesis in the *E. coli* Δ*bioH* strains.

Biotin biosynthesis has been proposed to be a promising target for antibiotic discovery given that it is required by all forms of life but can only be synthesized *de novo* by microorganisms and plants (Shapiro, 2013; Salaemae et al., 2016). Validation of biotin biosynthesis as a druggable antibacterial target is further supported by a number of genetic knockout studies with *Mycobacterium tuberculosis* and *Francisella novicida*. For instance, *M. tuberculosis* BioA and BioF have been shown to be essential for mycobacterial survival and virulence (Sassetti and Rubin, 2003; Woong Park et al., 2011). Deletion of *bioJ* in *F. novicida* showed that the gene was necessary for bacterial viability and replication in macrophage as well as for survival in mice (Feng et al., 2014). In particular, many inhibitors targeting biotin synthetic enzymes have been developed to effectively attenuate bacterial survival (Soares da Costa et al., 2012; Zlitni et al., 2013; Liu et al., 2017). Therefore, identifying genes essential for the biotin synthetic pathway may reveal a new valuable target for therapeutic interventions of *M. catarrhalis* infection.

In this study, we found that *M. catarrhalis* encodes all of the proteins required for assembly of the fused heterocyclic rings of biotin (Table S1). *M. catarrhalis* also encodes BioC in its biotin operon (Figure 1A) but no gene encoding a *bioH* homolog is present in the operon or genome, leading the possibility that *M. catarrhalis* must have a novel esterase to remove the methyl group of the methyl pimelyol-ACP introduced by its cognate BioC. We used a genetic complementation approach to isolate a gene encoding a protein having BioH function. This gave a new pimeloyl-ACP methyl ester esterase isozyme called BtsA, required to complete the biotin synthetic pathway in *M. catarrhalis*. BtsA was shown to play roles in bacterial invasion to lung epithelial cells and replication within macrophages. In summary, we provide the molecular mechanism of the biotin precursor biosynthesis as a *Moraxella* nutritional virulence factor.

## Materials and Methods

### Bacterial Strains, Plasmids, and Growth Conditions

Bacterial strains and plasmids used in this study are listed in Table 1. *E. coli* strains were grown at 37°C in Luria–Bertani (LB) medium (tryptone, 10 g/l; yeast extract, 5 g/l; NaCl, 10 g/l; pH 7.0). The biotin-free M9 minimal media for *E. coli* containing 0.05% vitamin-free Casamino Acids plus avidin (0.1 U/ml) and 0.2% glycerol as sole carbon source was used to test for biotin requirements. L-arabinose was added at a final concentration of 0.02%. *M. catarrhalis* strains were grown on brain heart infusion (BHI) plates at 37°C with 5% CO_2_ or in BHI broth with shaking at 37°C. The defined medium for *M. catarrhalis* containing S-715 mineral salts solution (Juni et al., 1986), 0.05% vitamin-free Casamino Acids and 1% DL-sodium lactate as sole carbon source was used to test for biotin requirements. Oligonucleotide primers (listed in Table S2) were synthesized and the cloned genes were verified by sequencing performed by GeneScript Co. (China). *M. catarrhalis* ATCC 25238 genomic DNAs was used as templates. Primers RS05165-F and RS05165-R were used to amplify *btsA* (*A4U55_05020*), and RS03745-F and RS03745-R for *A4U55_03600*. The PCR products were inserted into plasmid pBAD24M at sites NdeI and HindIII using T4 DNA ligase (NEB) to generate the plasmids, pBHK193 and pBHK194. Plasmid purification and PCR product purification were carried out according to the kit manuals (Tiangen Biotech Co., LTD, China).

**Table 1:** Bacterial strains and plasmids used in this study.

**Bacterial strains**	**Genotype**	**References** **or source**
***E. coli***		
DH5α	Δ(*argF-lac*)*U169* ϕ80d*lacZ58*(M15) Δ*phoA8 glnV44 deoR481 gyrA96 recA1 endA1 hsdR17*	Lab stock
BL21 (Tuner)	F^−^*ompT hsdS*_B_ (${\text{r}}_{\text{B}}^{-}$ ${\text{m}}_{\text{B}}^{-}$) *gal dcm lacY1*	Novagen
Rosetta™(DE3) pLysS	F^−^*ompT hsdS*_B_ (${\text{r}}_{\text{B}}^{-}$ ${\text{m}}_{\text{B}}^{-}$) *gal dcm* (DE3) pLysSRARE	Novagen
STL24	MG1655, Δ*bioH*	(Lin et al., 2010)
STL23	MG1655, Δ*bioC*	(Lin et al., 2010)
ER90	Δ*bioF bioC bioD*	(Choi-Rhee and Cronan, 2005)
***M. catarrhalis***		
ATCC 25238	Wild-type strain	(Jakobsson et al., 2016)
BHKS211	*M. catarrhalis* ATCC 25238, Δ*btsA*	This work
BHKS235	*M. catarrhalis* ATCC 25238, Δ*A4U55_03600*	This work
BHKS366	*M. catarrhalis* ATCC 25238, Δ*bioC*	This work
BHKS367	*M. catarrhalis* ATCC 25238, Δ*btsA::btsA*	This work
BHKS368	*M. catarrhalis* ACCT25238, IR*pssA*:: *catGC*	This work
BHKS376	*M. catarrhalis* ATCC 25238, Δ*bioC::bioC*	This work
BHKS449	*M. catarrhalis* ATCC 25238, Δ*btsA::BsbioI*	This work
**PLASMIDS**		
pQE-2	Amp^r^, T5 promoter-based expression vector	Qiagen
pBluescript SK(+)	Amp^r^, cloning vector	Stratagene
pBAD24M	Amp^r^, NcoI site of pBAD24 changed to an NdeI site, modified pBR322 origin	(Zhu et al., 2010)
pCM184	Km^r^, Allelic exchange vector	(Marx and Lidstrom, 2002)
pBHK193	Amp^r^, *A4U55_05020* (*btsA*) cloned into the NdeI and HindIII sites of pBAD24M	This work
pBHK194	Amp^r^, *A4U55_03600* cloned into the NdeI and HindIII sites of pBAD24M	This work
pBHK325	Amp^r^, *A4U55_05020* (*btsA*) cloned into the NdeI and HindIII sites of pQE-2	This work
pBHK384	Amp^r^, *A4U55_00945* (*bioC*) cloned into the NdeI and HindIII sites of pQE-2	This work
pBHK343	Amp^r^, *btsA* S117A of pBHK193	This work
pBHK344	Amp^r^, *btsA* D254A of pBHK193	This work
pBHK345	Amp^r^, *btsA* H287A of pBHK193	This work
pBHK348	Amp^r^, *btsA* S117A of pBHK325	This work
pBHK349	Amp^r^, *btsA* D254A of pBHK325	This work
pBHK350	Amp^r^, *btsA* H287A of pBHK325	This work
pBHK202	Amp^r^, Cm^r^; PCR-amplified *catGC* inserted between PstI; and BamHI sites of pBluescript SK(+)	(Jiang et al., 2019)
pBHK352	Km^r^, PCR-amplified McBtsAup inserted between EcoRI and KpnI sites plus PCR-amplified McBtsAdn inserted between ApaI and SacI sites of pCM184	This work
pBHK353	Km^r^, PCR-amplified McbioCup inserted between EcoRI and KpnI sites plus PCR-amplified McbioCdn inserted between ApaI and SacI sites of pCM184	This work
pBHK358	Amp^r^, PCR-amplified McUp inserted between KpnI and ApaI sites plus PCR-amplified McDn inserted between XbaI and SacI sites of pBHK202	This work
pBHK354	Amp^r^, Cm^r^; PCR-amplified *btsA* plus its promoter inserted between XhoI and SalI sites of pBHK358	This work
pBHK355	Amp^r^, Cm^r^; PCR-amplified *bioC* plus its promoter inserted between XhoI and SalI sites of pBHK358	This work
pBHK356	Amp^r^, Cm^r^; PCR-amplified Bs*bioI* plus *bio* operon promoter inserted between XhoI and SalI sites of pBHK358	This work

### Construction of *M. catarrhalis* Mutants

The *M. catarrhalis* Δ*btsA* strain (BHKS211) was constructed using the allelic exchange vector pCM184 (Marx and Lidstrom, 2002). The PCR amplified upstream and downstream homologous arm of the target gene *A4U55_05020* using two primer sets RS05165UP-EcoRI-F/RS05165UP-KpnI-R and RS05165Dn-ApaI-F/RS06165Dn-SacI-R were inserted into the vector pCM184 successively to generate plasmid pBHK352. This plasmid was then introduced into *M. catarrhalis* ATCC 25238 by natural transformation via allelic exchange. Colonies (Δ*btsA*) were isolated on BHI agar plates supplemented with kanamycin (25 μg/ml). By the same method, the Δ*bioC* strain (BHKS366) was constructed by introducing the plasmid pBHK353 that harboring the upstream and downstream homologous arm of the target gene *bioC* amplified by two primer sets BioCUP-EcoRI-F/BioCUP-KpnI-R and BioCDn-ApaI-F/BioCDn-SacI-R, and followed by screening successful double cross recombination events on kanamycin plates. The deletion of *M. catarrhalis bioC* or *btsA* in the genome of the mutant strain was confirmed by PCR using appropriate primers (Figure S1), followed by sequencing of the PCR products.

### Construction of *M. catarrhalis* Complementation Strains

The complemented Δ*btsA*::*btsA* strain (BHKS367) was constructed by inserting a wild-type copy of the *btsA* gene in the locus between two convergent expression gene *pssA* and *MCR_1955* where is untranslated regions of the *M. catarrhalis* chromosome. Firstly, the upstream and the downstream untranslated regions between *pssA* and MCR_1955 were amplified by PCR using two primer pairs (McUp-KpnI-L/McUp-ApaI-R and McDn-XbaI-L/McDn-SacI-R), respectively, and then ligated to pBHK202 (Jiang et al., 2019) to generate pBHK358. This plasmid carrying a sandwich fusion in which the *catGC* cassette (encoding chloramphenicol resistance gene, about 960 bp) was flanked by the upstream and the downstream untranslated regions. The application of this complementation system was confirmed by the construction of the *M. catarrhalis* IR*pssA*::*catGC* strain (BHKS368) by natural transformation via allelic exchange and observation with its growth phenotype and biotin requirement. Note that this strain has no effect on growth and biotin synthesis (Figure S2). The *btsA* gene plus its own promoter region was amplified by PCR with the primer set McBtsA-XhoI-F/McBtsA-SalI-R and cloned into the XhoI and SalI sites (upstream of *catGC* cassette) of pBHK358 to generate pBHK354. This plasmid was then introduced into *M. catarrhalis* Δ*btsA* (BHKS211) by natural transformation and successful double cross recombinants Δ*btsA*::*btsA* (BHKS367) were screened on BHI agar plates supplemented with kanamycin (25 μg/ml) and chloramphenicol (5 μg/ml). The complementation of *btsA* in the genome of the complemented strain was confirmed by PCR using appropriate primers followed by sequencing of the PCR products.

The complemented *M. catarrhalis* Δ*bioC*::*bioC* strain (BHKS376) was also constructed by the same methods as described above. Instead, the *bioC* coding region and its *bioAFCD* operon promoter region were amplified by primer set McBioC-3/McBioC-SalI-R and McBioC-XhoI-F/McBioC-2, respectively, and then fused together to one fragment by fusion PCR using primer McBioC-XhoI-F/McBioC-SalI-R. The fused fragment was then inserted into the pBHK358 at XhoI and SalI sites to generate pBHK355, whicht was then introduced into *M. catarrhalis* Δ*btsA* (BHKS211) by natural transformation. The complemented *M. catarrhalis* Δ*bioC*::*bioC* strain (BHKS376) was screened on kanamycin (25 μg/ml) and chloramphenicol (5 μg/ml) supplemented plates and confirmed by PCR using appropriate primers and sequencing.

The *M. catarrhalis* Δ*btsA*::*BsbioI* strain (BHKS449) was also constructed as described above. The *Bacillus subtilus bioI* gene was amplified from *B. subtilis* genomic DNA using primers BsBioI-3/BsBioI-SalI-R while its promoter region was amplified from its upstream *bio* operon promoter region with primers BsBioI-XhoI-F/BsBioI-2. These two PCR fragments were then ligated together by fusion PCR using primers BsBioI-XhoI-F/BsBioI-SalI-R to assure the *BsbioI* gene can be transcribed on its own promoter in the *M. catarrhalis* strain. Hence the fused fragment was then inserted into the pBHK358 at XhoI and SalI sites to generate pBHK356. The following construction and confirmation was the same as described above.

### Protein Expression and Purification and Chemical Cross-Linking Assays

The *btsA* gene was amplified from *M. catarrhalis* ATCC 25238 genomic DNAs and inserted into vector pQE-2 to give plasmid pBHK325 which encodes BtsA with a N-terminal hexahistidine (His)-tag. BtsA was expressed in Rosetta (DE3) pLysS grown at 37°C in LB medium. At an OD600 of 0.8, the cultures were induced with 0.05 mM isopropyl-β-D-thio-D-galactoside (IPTG) and grown at 30°C for an additional 6 h prior to harvest. The cells were collected, resuspended in lysis buffer (50 mM sodium phosphate, 300 mM NaCl, 10 mM imidazole, 1 mM dithiothreitol, pH 8.0), lysed by ultrasonication and centrifuged (20,000 rpm/min, 40 min). The clarified bacterial supernatant was loaded onto a nickel-ion affinity column (Qiagen). The column was washed with wash buffer (50 mM NaH_2_PO_4_, 300 mM NaCl, 40 mM imidazole, 1 mM dithiothreitol [pH 8.0]) to remove contaminating proteins, and the His-tagged BtsA protein was eluted in the same buffer (elution buffer) containing 200 mM imidazole. The protein was concentrated by ultrafiltration (10 kDa cut-off) and exchanged into sodium phosphate buffer (50 mM NaH_2_PO_4_, 150 mM NaCl, 1 mM dithiothreitol, pH 8.0). The protein purity was visualized by gradient SDS-PAGE (12%) and further confirmed by liquid chromatography quadruple time-of-flight (qTOF) mass spectrometry of tryptic peptides (performed by University of Nanjing Mass Spectrometry Laboratory). To further test the solution structure of BtsA, chemical cross-linking with ethylene glycol bissuccinimidylsuccinate (Pierce) was performed as described previously (Bi et al., 2012). In each chemical cross-linking reaction (20 μl in total), the purified BtsA protein (~10 mg/ml) was separately mixed with different concentrations of cross-linker (0, 0.5, 2, 10, 50, 250, and 1,250 μM), and incubated for 30 min at room temperature. All the reaction products were separated using gradient SDS-PAGE. Recombinant BioC from *M. catarrhalis* was purified from an overproducing *E. coli* Rosetta(DE3) strain transformed with pBHK384 carrying the *A4U55_00945* gene, as described above.

### Site-Directed Mutagenesis of *btsA*

Plasmids pBHK343, pBHK344, and pBHK345 each carrying a single mutation within the BtsA coding sequence were obtained using the QuickChange mutagenesis kit with pBHK193 as the PCR template. The primers used in PCR and mutagenesis are listed in Table S2. The constructed plasmids were transformed into *E. coli* DH5α. The mutations were verified by DNA sequencing. These three BtsA mutant plasmids were then introduced into *E. coli* Δ*bioH* strain STL24 for complementation ability test. Plasmids pBHK348, pBHK349, and pBHK350 each carrying a single mutation were obtained using the QuickChange mutagenesis kit with pBHK325 as the PCR template.

### Esterase Activity Assays

The reactions in 10 μl total volume contained 100 mM sodium phosphate (pH 7.8), 100 μM pimeloyl-ACP methyl ester, 1 mM DTT and different concentrations of BtsA (0.1, 0.3, 1, 3, and 5 μg) or its mutant derivatives. A premix of buffer and the ACP substrate, lacking BtsA, was incubated at 37°C for 2 min. The hydrolysis reaction was initiated by adding BtsA or (its mutant derivatives) and incubated at 37°C for 30 min. The reaction samples were loaded into 18% PAGE gel containing 2.5 M urea and then ran at 100 V for 2.5 h. The pimeloyl-ACP methyl ester was synthesized using *Vibro harveyi* acyl-ACP synthetase AasS (Lin et al., 2010) and *E. coli* holo-ACP as described previously (Cronan and Thomas, 2009).

### *In vitro* DTB Synthesis

An *in vitro* system that utilized crude extracts of strain STL24 *(E. coli* Δ*bioH*) was reconstituted to test the potential role of BtsA in biotin biosynthesis, as described by Lin et al. (2010) with some modifications. The cell-free extracts from *E. coli* Δ*bioH* were prepared as described previously (Lin et al., 2010). The production of DTB was visualized using the biotin auxotrophic strain ER90 (Δ*bioF bioC bioD*)-based biotin bioassay. This assay allows *in vitro* conversion of ACP-bound substrate into DTB using enzymes in cell-free extracts. A 100 μl reaction in assay buffer contained 1 mg of Δ*bioH* cell-free extract protein, 1 μmol MgCl_2_, 0.5 μmol dithiothreitol, 0.01 μmol pyridoxal-5′-phosphate, 50 μg pimeloyl-ACP methyl ester, 0.1 μmol L-alanine, 0.1 μmol KHCO_3_, 0.1 μmol NADPH, 0.1 μmol ATP, 0.1 μmol glucose-6-phosphate, and 0.1 μmol SAM. A similar *in vitro* DTB synthesis reaction for testing the role of *M. catarrhalis* BioC was performed with 1 mg *E. coli* Δ*bioC* cell-free extract protein, 50 μg malonyl-ACP, 2 μg BioC, and other cofactors as above. The reactions were incubated at 37°C for 5 h and quenched by immersion in boiling water for 10 min. DTB production was bioassayed as follows. *E. coli* strain ER90 was grown in 5 ml of glucose M9 minimal medium containing 2 nM biotin at 30°C overnight. The cells were washed with M9 medium and subcultured in 100 ml of glucose minimal medium at 37°C for 5 h to starve the cells for biotin. The cells were collected by centrifugation, washed again in M9 medium and mixed into 100 ml of glucose minimal agar containing the redox indicator 2,3,5-triphenyl tetrazolium chloride (0.1%, w/v) to a final OD at 600 nm of ~0.05. About 15–20 ml of the mixture was poured into Petri dishes sectored with plastic walls to prevent cross-feeding. A 6 mm paper disk (BBL) was placed upon the agar, and the disk was spotted with 10 μl of a reaction to be tested. After incubation of the plates at 30°C overnight, growth of strain ER90 was visualized as a red deposit of formazan. The malonyl-ACP was enzymatically synthesized from malonyl-CoA purchased from Sigma using *Bacillus subtilus* Sfp phosphopantetheinyl transferase (Quadri et al., 1998), as described previously (Massengo-Tiasse and Cronan, 2008).

### Assessment of Adherence and Invasion of Respiratory Epithelial Cells

Quantitative adherence and invasion assays were performed with A549 cells (human type II alveolar lung epithelium) grown in 1640 media (Gibco) plus 10% fetal bovine serum as previously described (Murphy et al., 2013). This adherence and invasion experiments in the present study use an MOI of 10. Briefly, 2 × 10^5^ A549 cells were seeded into each well of a 24 well tissue culture plate and incubated for ~24 h when cells showed confluent growth. Cells were inoculated with BHI broth-grown log phase bacteria and the plates were centrifuged at 1,000 rpm for 10 min at room temperature to facilitate contact between bacteria and A549 cells. Plates were incubated for 3 h at 37°C. Non-adherent cells were removed by gently washing the wells 3 times with PBS. To quantify adherent cells, 200 μl of trypsin (0.25%) was added to each well and plates were incubated at 37°C for 10 min to remove adherent cells. A 300 μl volume of 0.1% saponin was added to each well, and transferred into 1.5 ml tube and vortexed vigorously, counted on plates with dilution after overnight incubation. Adherence was measured as colony forming units (CFU) per ml. Results of assays with the mutants (CFU/ml) were expressed as a percent of the result with wild type (CFU/ml) that was performed simultaneously. Each experiment was repeated three independent times.

To measure bacterial invasion ability, A549 cells and strains were co-incubated 3 h. Non-adherent cells were removed by washing and further killed by gentamicin (100 μg/ml) for 1 h incubation at 37°C. Epithelial cells were recovered with trypsin and lysed with 1% saponin as described above, and then plated in duplicate. The results were represented by the percentage of the number of viable mutant bacteria (CFU/ml) accounting for wild type bacteria number (CFU/ml). Each experiment was repeated three independent times and the average and standard deviation were calculated.

### Macrophage Survival Assays

Bacterial survival was quantitated using modified gentamicin protection assays as previously described (Brett et al., 2008). Briefly, RAW 264.7 macrophages (1~2 × 10^5^ cells) were pre-seeded in a 24-well plate per well and incubated at 37°C with 5% CO_2_ for 15–20 h in DMEM plus 10% FBS serum culture medium. Overnight bacterial cultures were grown on the agar at 37°C and resuspended with DMEM medium. Bacterial cultures were used to infect macrophages at an MOI of 100. Upon infection, plates were centrifuged for 5 min at 500× g and were incubated for 30 min at 37°C with 5% CO_2_. Then phagocytosis was stopped by washing three times with PBS to remove non-adherent bacteria, followed by 1 h of additional gentamicin (100 μg/ml) treatment to kill the adherent bacteria. The total viable intracellular bacteria were then estimated on BHI agar plates after cell lysis (1% w/v saponin), set as T0 post-infection. For the post-infection measurement after T0, infected cells were further incubated in DMEM medium plus 10% FBS and survival bacteria within the macrophages were released at the indicated time point during 12 h post-infection and quantified by plating serial dilutions on BHI agar plates and enumerating colony counts. Data are represented as CFU/ml. Each experiment was repeated three independent times and the average and standard deviation were calculated.

### Bioinformatic Analyses

The multiple alignments of BtsA proteins were conducted using the Clustal Omega program (https://www.ebi.ac.uk/Tools/msa/clustalo/), and final output was processed by the ESPript 3.0 server (http://espript.ibcp.fr/ESPript/cgi-bin/ESPript.cgi). Minimum evolution phylogenetic trees were inferred with Mega6 program. The statistical robustness and reliability of the branching order within each phylogenetic tree were confirmed with a bootstrap analysis using 1,000 replicates. Sequences to be analyzed were retrieved from the NCBI microbial protein database http://www.ncbi.nlm.nih.gov/sutils/genom_table.cgi). The amino acid sequence of BtsA was submitted to the CPHmodels 3.0 Server (http://www.cbs.dtu.dk/services/CPHmodels), generating a PDB file of the modeled structure, which searches for a reasonable template of known structure.

## Results

### *M. catarrhalis btsA*, a Novel Biotin Biosynthesis Gene

**Figure 2:**
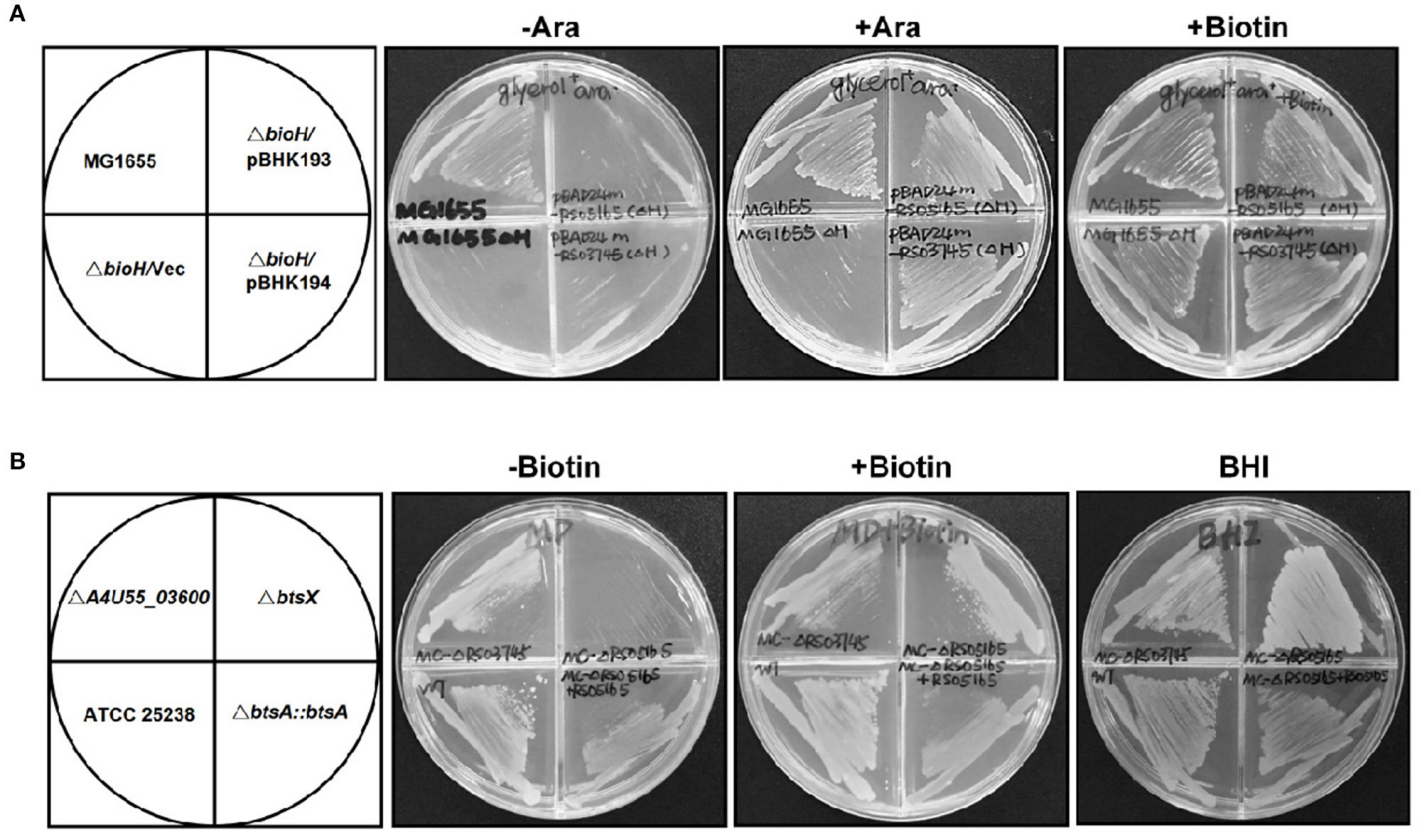
Identification of *btsA* as an essential biotin synthesis gene *in vivo*. **(A)** Expression of *M. catarrhalis btsA* (*A4U55_05020*) allows growth of the *E. coli* Δ*bioH* strain on biotin-free medium. Transformants of strain STL24 (*E. coli* Δ*bioH*) were grown at 37°C on biotin-free medium with glycerol as carbon source. Growth in the absence of biotin was tested in either the presence or the absence of 0.02% arabinose (Ara). Glycerol gives basal expression of BtsA whereas arabinose gives induced expression. The strains tested were: MG1655 (wild type strain), and STL24 carrying plasmid pBHK193 encoding *btsA*, pBHK194 encoding *A4U55_03600* or the empty plasmid pBAD24M (Vec). Growth on plates containing 4 nM biotin was used as a positive control. **(B)** *M. catarrhalis* requires *btsA* for growth. Growth phenotypes of various *M. catarrhalis* strains were observed on the defined plates with or without biotin (10 nM) supplementation. The plates were incubated at 37°C for 2 days. The strains tested were: ATCC 25238 (wild type strain), Δ*A4U55_03600*, Δ*btsA*, and b*BtsA::btsA*. Growth on BHI plates was used as a positive control.

Analysis of the *M. catarrhalis* genome revealed that it contains four genes encoding homologs of *E. coli* BioA, BioF, BioC, and BioD that are clustered in an operon, but the *bioB* gene is remote from the cognate *bioAFCD* operon (Figure 1A). Intriguingly, six known pimeloyl-ACP methyl ester esterase isozymes seemed to be absent in all the examined *Moraxella* species, indicating functional replacement by an unknown novel enzyme. Note that three ORFs of *M. catarrhalis* ATCC 25238 (*A4U55_03350, A4U55_03600*, and *A4U55_05020*) were annotated as putative α*/*β hydrolases. To test if expression of these three genes could provide the missing biotin biosynthesis gatekeeping enzyme, we assayed complementation of an *E. coli* Δ*bioH* biotin auxotrophic strain STL24. A derivative of arabinose inducible vector pBAD24M carrying each putative gene was transformed into the Δ*bioH* strain and the transformants were streaked on the biotin-free minimal media with or without arabinose. As shown in Figure 2A, plasmid-borne expression of *A4U55_03600* or *A4U55_05020* allowed robust growth of the Δ*bioH* strain on plates that lacked biotin, whereas the empty vector failed to support detectable growth. Strong growth was only seen in the presence of arabinose (the inducer of the araBAD promoter), indicating that induced expression is required. Note that *A4U55_03350* has no such complementary ability (data not shown). As a positive control, in the presence of 4 nM biotin the strains that carried either genes or the empty vector both grew well (Figure 2A).

In order to determine if these two putative genes that participate in the biotin synthesis of *M. catarrhalis in vivo*, we constructed the *A4U55_03600* and *A4U55_05020* knockout mutants and complemented the mutants by double crossover homologous recombination (Figure S1), respectively. Growth phenotypes were evaluated on the biotin-free minimal medium. As shown in Figure 2B, without biotin addition, the Δ*A4U55_05020* strain failed to support detectable growth and complementation restored the growth. As expected, when biotin was added to the plate, it rescued growth defect of the Δ*A4U55_05020* strain indicating that this mutant is a biotin auxotrophic strain. In contrast, a robust growth was observed in the Δ*A4U55_03600* strain supplemented with or without biotin, which is similar with the wild-type strain (Figure 2B). Although the above complementation assay studies indicated that both *A4U55_03600* and *A4U55_05020* functionally replace *E. coli bioH*, the *A4U55_05020* gene was the only one required for the biotin synthesis of *M. catarrhalis in vivo*. Given that A4U55_05020 and other BioH paralogues lack sufficient sequence similarity for alignment, we have designated *A4U55_05020* as *btsA*. In addition, we found that deletion of *A4U55_00945* (*bioC*) also caused a biotin auxotrophic phenotype, and the complementation of *bioC* gene expression rescued the mutant growth on the biotin-free plate (Figure S2). Taken together, all these observations suggest that *btsA* and *bioC* are key partners that participate in the biotin synthesis pathway in *M. catarrhalis*.

### BtsA Recognizes the Pimeloyl-ACP Methyl Ester *in vitro*

**Figure 3:**
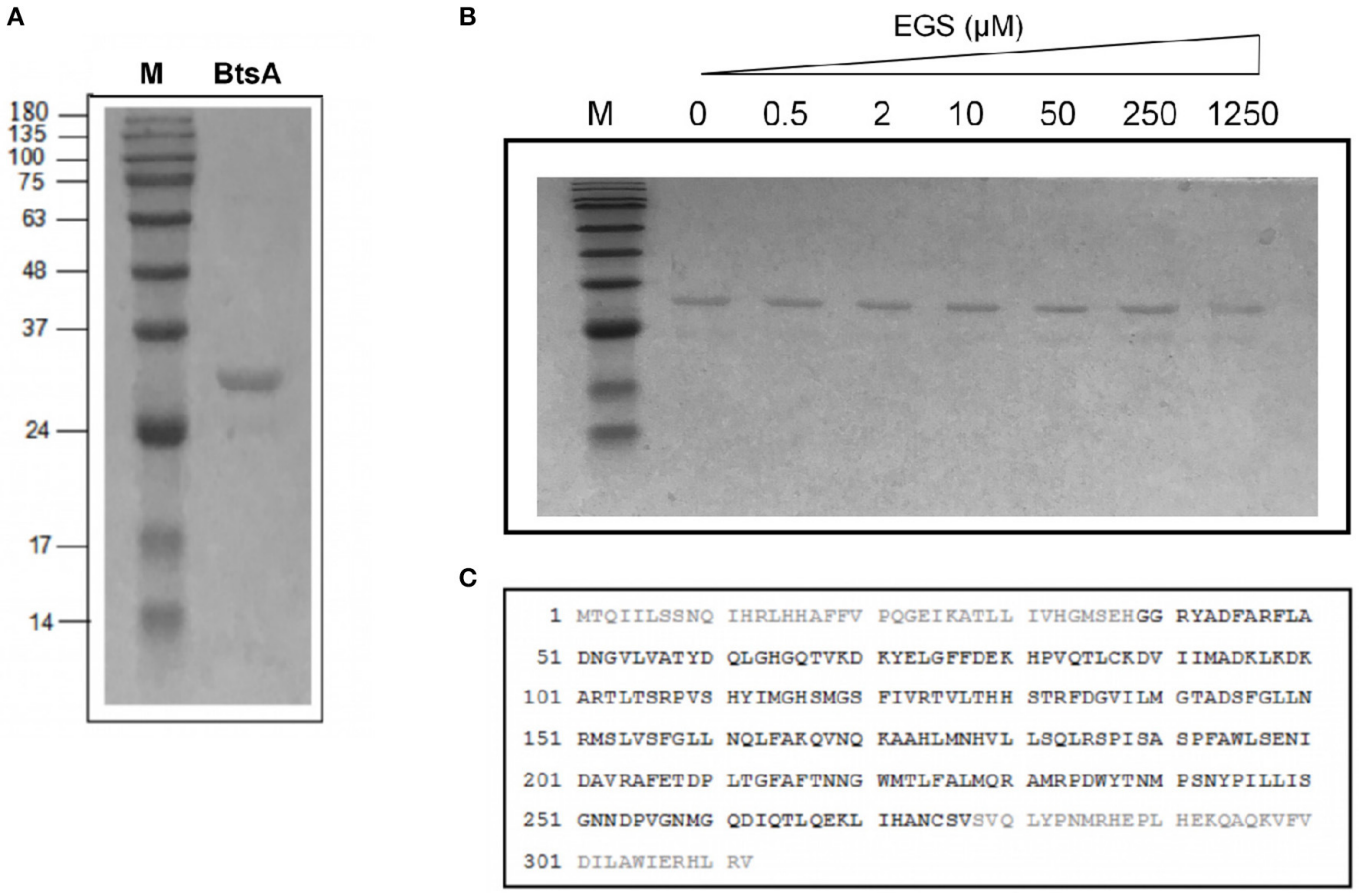
Purification and structural characterization of BtsA. **(A)** SDS-PAGE analysis of the purified BtsA. The apparent molecular weight of His-tagged BtsA is about 30 kDa. M, Molecular weight. **(B)** Mass spectrometric identification of BtsA. The matching peptides are given in bold. **(C)** Chemical cross-linking analyses of BtsA where EGS denotes ethylene glycol bissuccinimidylsuccinate. The samples of BtsA reaction mixtures with EGS at different concentrations were separated by 10% SDS-PAGE.

To determine if BtsA functions as an esterase that converts pimeloyl-ACP methyl ester into pimeloyl-ACP *in vitro*, we purified the protein and assayed its *in vitro* activity. The recombinant BtsA with an N-terminal hexahistidine tag was readily expressed in *E. coli* and purified by Ni^2+^-chelating chromatography. The SDS-PAGE and chemical cross-linking results showed that the purified BtsA molecular weight is 30 KDa and exists as a typical monomeric structure of α*/*β-hydrolase in solution (Holmquist, 2000) (Figures 3A,B). Liquid chromatography mass spectrometry of tryptic peptides validated the identification of the recombinant protein with 76% coverage of the peptides predicted from the DNA sequence (Figure 3C).

**Figure 4:**
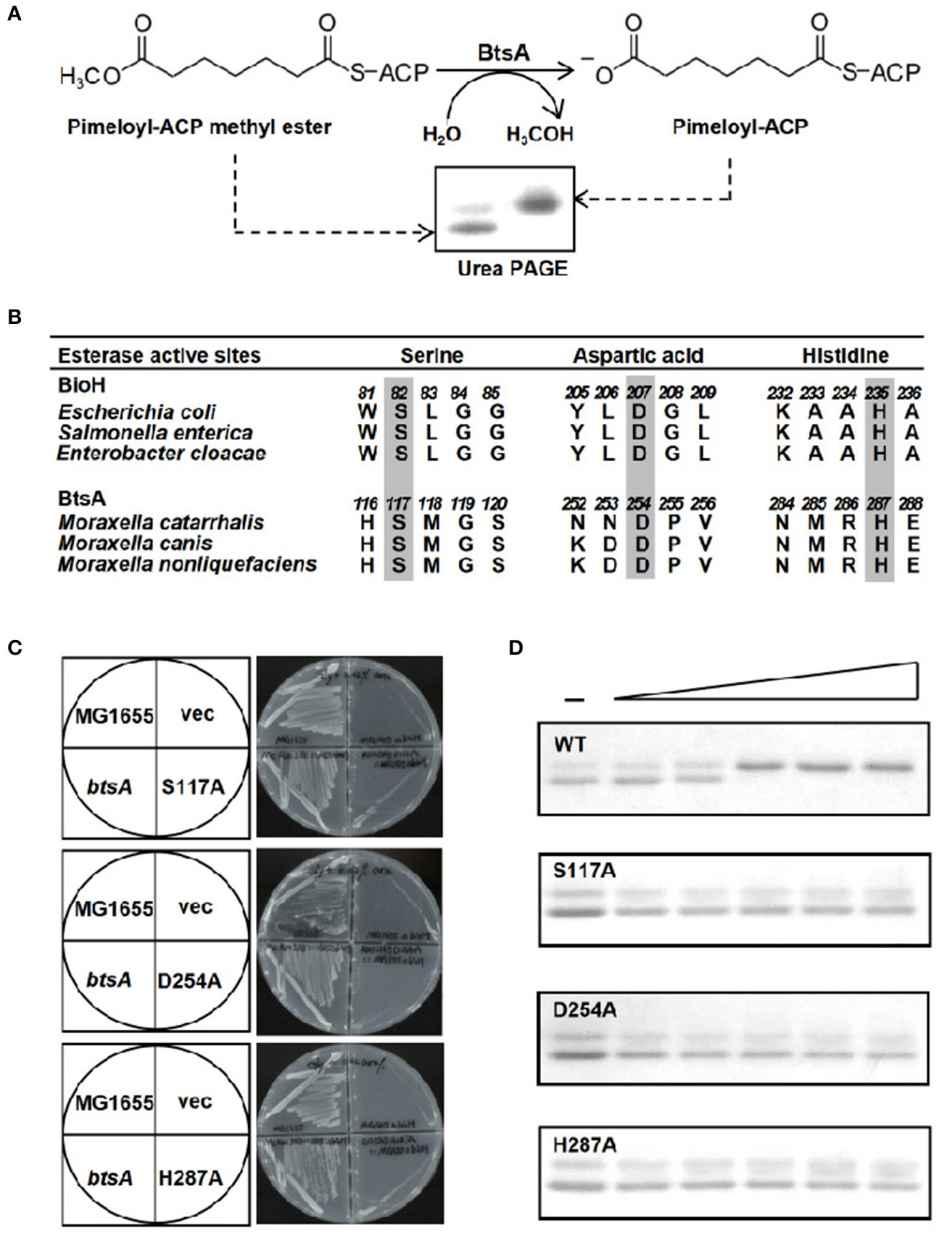
The BtsA protein cleaves the ester group of pimeloyl-ACP methyl ester. **(A)** Schematic diagram of the enzymatic reaction catalyzed by BtsA. The enzymatic reactions were performed at 37°C and contained 100 μM pimeloyl-ACP methyl ester as substrate. The hydrolysis product pimeloyl-ACP migrates more slowly than the substrate in a destabilizing urea-PAGE system. **(B)** Comparison of the putative active sites of BtsA with the catalytic residues of *E. coli* biotin synthetic esterases. The key residues are in gray. **(C)** *In vivo* functional analyses of the triad residue BtsA mutant proteins. The wide type and transformants of strain STL24 (*E. coli* Δ*bioH*) were grown at 37°C on biotin-free medium. Growth was tested in either the presence or the absence of arabinose. The strains tested were: STL24 carrying plasmids pBHK193, pBHK343, pBHK344, and pBHK345 encoding wild type *btsA*, and one of the mutant derivatives, S117A, D254A, or H287A, respectively. The vector plasmid (vec), pBAD24M was also included. **(D)** *In vitro* functional analyses of the triad residue BtsA mutant proteins. Enzymatic activities of BtsA and the single mutant S117A, D254A, or H287A proteins were assayed by the conformationally sensitive electrophoretic mobility shift assay. Minus denotes no addition of BtsA (or a mutant protein) whereas the triangle on the right hand represents the protein levels in an inverse dilution series (0.1, 0.3, 1, 3, and 5 μg). The enzymatic reaction (10 μl total volume) contained 100 μM pimeloyl ACP methyl ester. The reaction mixture was separated using 18% PAGE containing 2.5 M urea.

The activity of BtsA was determined using a conformationally sensitive gel electrophoretic mobility shift assay. As previously reported, pimeloyl-ACP migrated more slowly than the substrate pimeloyl-ACP methyl ester because the loss of hydrophobic methyl easter group changed the charge of carboxyl group of ACP and hence expanded the ACP moiety (Shapiro et al., 2012; Feng et al., 2014). As shown in Figure 4A, BtsA catalyzed hydrolysis of the methyl ester bond of pimeloyl-ACP methyl ester and the product pimeloyl-ACP moved slowly than the substrate. To provide further evidence that BtsA recognizes the pimeloyl-ACP methyl ester *in vitro*, we constructed strain BHKS449, a Δ*btsA* derivative strain carrying the *Bacillus subtilis bioI* gene cleaving the C7-C8 bonds of acyl-ACPs to give pimeloyl-ACP (Stok and De Voss, 2000; Cryle and Schlichting, 2008). As expected, the expression of BsBioI completely recovered the growth of the Δ*btsA* strain on biotin-free medium (Figure S3), suggesting McBioF could accept pimelate-ACP as a substrate to initiate completion of the fused heterocyclic rings of biotin. These results indicated that BtsA recognized and hydrolyzed the same substrate as *E. coli* BioH *in vitro*, in excellent accord with the *in vivo* complementation data.

### A Conserved Catalytic Ser-His-Asp Triad Is Essential for BtsA Activity

The known pimeloyl-ACP methyl ester esterase isozymes have been shown to employ a α*/*β-hydrolase catalytic domain with the conserved (Ser-Asp-His) catalytic triad to function as esterases (Shapiro et al., 2012; Feng et al., 2014; Bi et al., 2016). To further characterize if BtsA has similar active triad residues, we performed sequence alignment of BtsA homologs from different *Moraxella* species and predicted a catalytic triad consisting of Ser117, His254, and Asp287 (Figure 4B, Figure S4). To test function of the putative triad we constructed genes encoding three mutant BtsA proteins, S117A, D254A, and H287A in the pBAD24M expression vector by site-directed mutagenesis. As shown in Figure 4C, compared to the robust growth of wild-type *E. coli* strain MG1655 and the Δ*bioH* strain carrying *btsA*, the expression of each BtsA mutants failed to allow growth of the Δ*bioH* strain on biotin-free plates. The significance of these three amino acid residues was also verified by protein purification (Figure S5) and esterase hydrolysis reaction assays *in vitro*, and all mutant enzymes failed to cleave pimeloyl-ACP methyl ester (Figure 4D). Taken together, it is clear that *M. catarrhalis* BtsA is a member of α*/*β-hydrolase with a conserved Ser-His-Asp triad catalytic triad.

### BtsA Is Required for Desthiobiotin Synthesis

**Figure 5:**
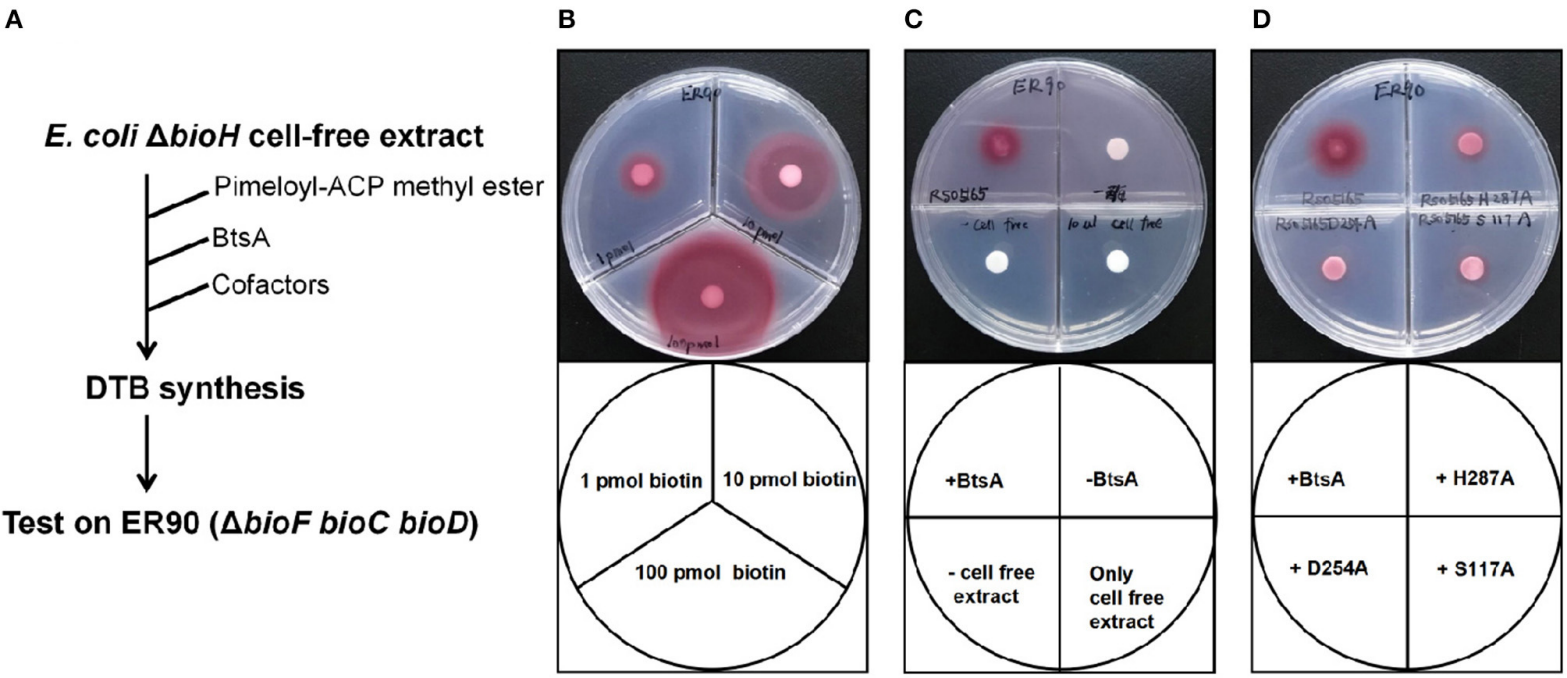
Bioassay of BtsA function in the overall biotin synthetic pathway. **(A)** Scheme of the *in vitro* DTB synthesis system of BtsA. **(B)** Very low (pmol) levels of biotin supported growth of the biotin auxotrophic strain ER90 (Δ*bioF bioC bioD*) on biotin-free minimal medium. **(C)** Restoration of DTB synthesis to the Δ*bioH* extract by addition of pimeloyl-ACP methyl ester and BtsA. The upper left quadrant contained BtsA proteins and all components required for DTB synthesis. The upper right quadrant reactions contained all components required for DTB synthesis except BtsA whereas the samples spotted on the lower left quadrants lacked strain STL24 (*E. coli* Δ*bioH*) cell extract, and lower right quadrant only contained cell free extract from STL24. **(D)** The mutant BtsA derivatives (S117A, D254A, or H287) did not restore DTB synthesis to the Δ*bioH* extract.

To further test the role of BtsA in biotin synthetic pathway, the reconstitution of desthiobiotin (DTB) bioassays were carried out based on the *in vitro* system previously developed, which converts malonyl-ACP to DTB using crude extracts of *E. coli* Δ*bioH* strain and the biotin auxotroph strain ER90 (Δ*bioF* Δ*bioC* Δ*bioD*) (Manandhar and Cronan, 2017). Growth of the indicator strain ER90 is visualized by deposition of a red formazan deposit (del Campillo-Campbell et al., 1979). The DTB bioassays can reliably detect 1 pmol of biotin or DTB production (Figure 5A). As shown in Figure 5B, addition of pimeloyl-ACP methyl ester to the crude extracts gave no detectable growth for ER90, whereas good growth was seen upon addition of purified BtsA to the reactions. In contrast, the incubation containing any of the mutant BtsA proteins gave no DTB synthesis (Figure 5C). This is also the case for *M. catarrhalis* BioC. An enlarged red zone was observed when purified BioC (Figure S6) was added into the reactions with the crude extracts prepared from the *E. coli* Δ*bioC* strain and the substrate malonyl-ACP (Figure S7). In contrast, no DTB synthesis was detected when the incubations contained any of the mutant BtsA proteins (Figure 5D). Thus, these results clearly demonstrated the physiological roles of BtsA and BioC in bacterial DTB biosynthesis.

### BtsA Plays Roles in Invasion of Respiratory Epithelial Cells and Intracellular Survival Within Macrophages

**Figure 6:**
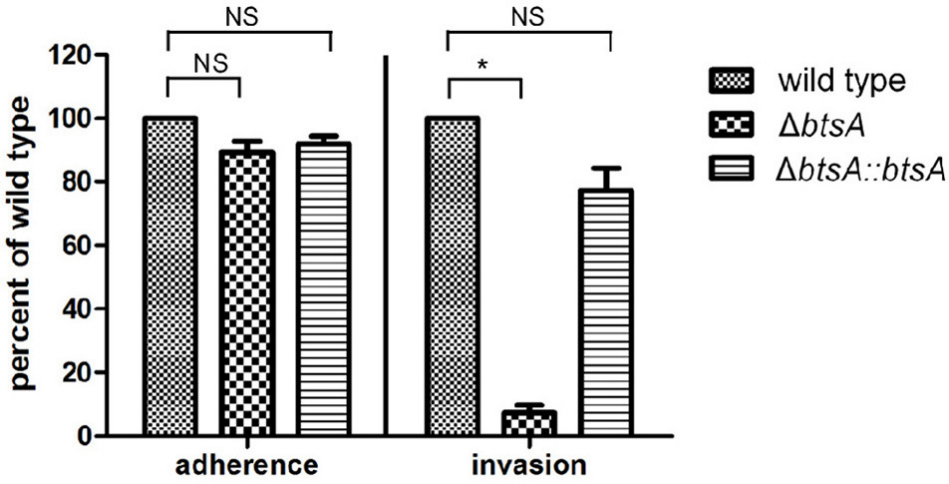
Adherence and invasion assays with the A549 respiratory epithelial cell line with *M. catarrhalis* strains. The cells were infected and, after 30 min for adherence and 3 h for invasion, total cell-associated bacteria or intracellular bacteria, respectively, were quantitated by dilution plating. Data are represented as means ± SD of three independent experiments. *P < 0.05.

Adhesion and invasion of *M. catarrhalis* to epithelial cells is critical for its pathogenesis (Verduin et al., 2002). To assess the role of BtsA in adherence to and invasion of human respiratory epithelial cells, quantitative adherence and invasion assays were performed on the type II alveolar cell lines (A549). As shown in Figure 6, adherence of the Δ*btsA* mutant was as efficient as that of the wild-type strain and the complemented strain. To determine if BtsA is required for efficient invasion, A549 cells were infected with each strain and following a 3 h incubation, cells were washed, incubated with gentamicin for an additional 2 h, and then lysed, serially diluted, and plated to determine the proportion of invading bacteria. The Δ*btsA* mutant showed statistically significant reduced invasion of epithelial cells compared to wild type (Figure 6), indicating that BtsA is required for efficient invasion of human respiratory epithelial cells *in vitro*. Note that complementation restored its invasion ability. In addition, impaired invasion ability was also observed with the Δ*bioC* strain (Figure S8). Thereafter, although *M. catarrhalis* biotin auxotroph has no effect on bacterial adherence of epithelial cells, it could effectively inhibit invasion and represent an evidence linking biotin synthesis to bacterial invasion and virulence.

**Figure 7:**
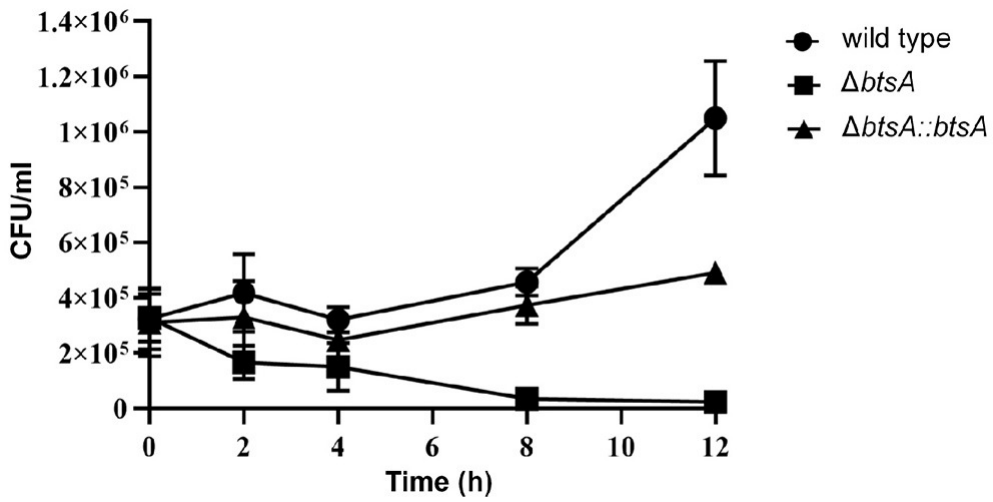
Survival and replication of *M. catarrhalis* strains in macrophages. The RAW 264.7 cells were infected with *M. catarrhalis* ATCC 25238 (solid circle), the Δ*btsA* strain (solid square) and the complemented strain Δ*btsA::btsA* (solid triangle) at an MOI of 100, and intracellular loads of bacteria were enumerated at indicated time points postinfection. Data are represented as means ± SD of three independent experiments.

Macrophages are key components of the innate immune system involved in orchestrating host defense against microbial infections. We then performed classical gentamicin protection assays using the murine macrophage cell line RAW 264.7, to determine survival of the wild-type and Δ*btsA* strains within macrophages. As shown in Figure 7, by 12 h post-infection the wild-type strain readily proliferated to high numbers from 3 × 10^5^ CFU/ml to 1 × 10^6^ CFU/ml. In contrast, once inside macropahges, the number of viable Δ*btsA* strains gradually decreased significantly and 12 h after post-infection, the viable cell number of the Δ*btsA* strain was 45-fold lower than that of the wild-type strain (Figure 7). Note that the complemented Δ*btsA::btsA* strain could partially reversed the replication defects within the RAW 264.7 cells, illustrating the physiological importance of BtsA to the intracellular survival of *M. catarrhalis* within macrophages. These results together with bacterial invasion assays indicate a role for biotin biosynthesis in *M. catarrhalis* virulence.

### *Moraxella* Species Encode a New Class of Carboxylesterase Paralogue

**Figure 8:**
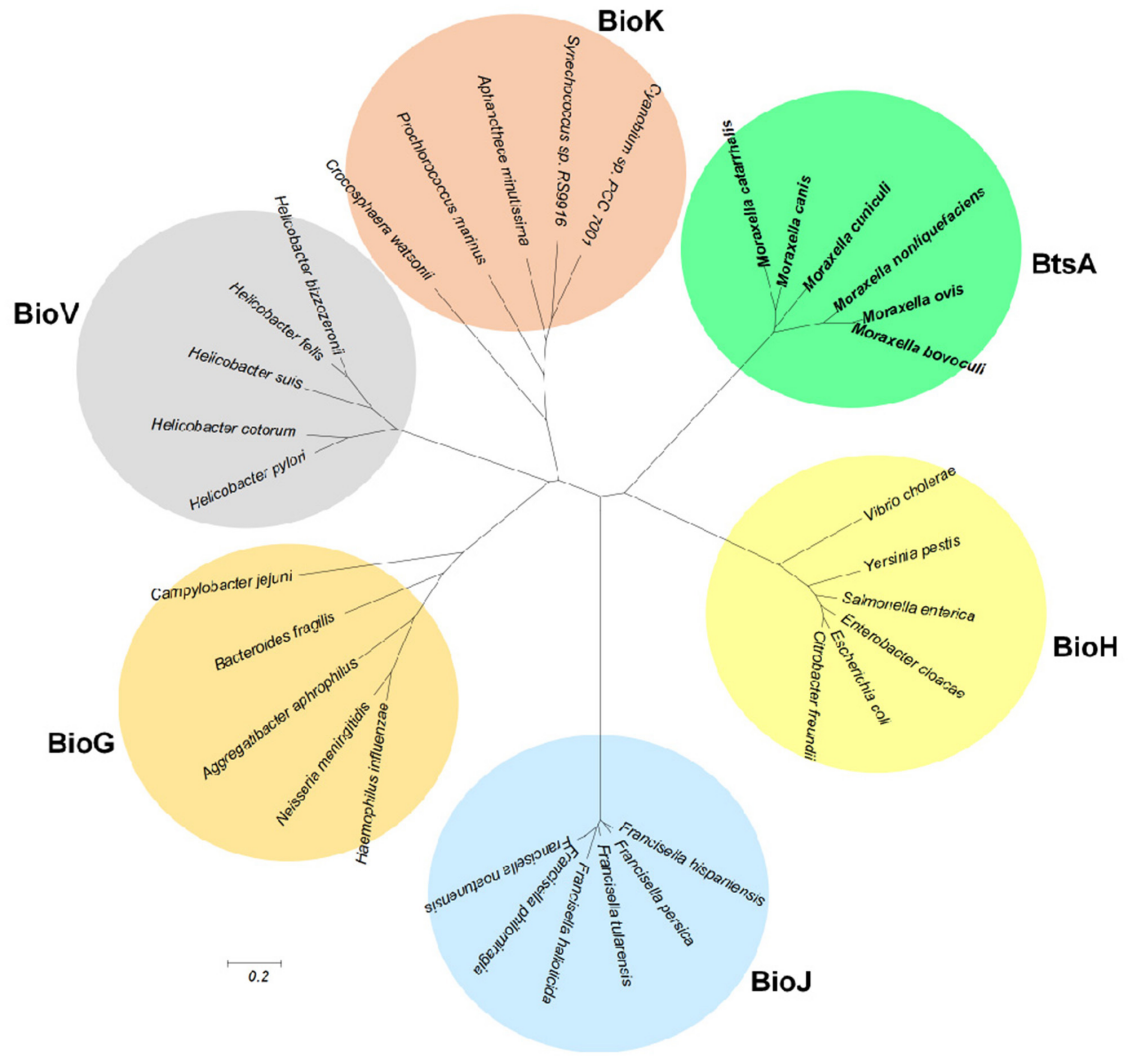
Phylogeny of the bacterial pimeloyl-ACP methyl ester esterases. Phylogenetic analyses were conducted by the minimum-evolution method using MEGA6. BtsA from *Moraxella* species are in bold.

Although BtsA is predicted to be a member of the α,β-hydrolase family and shares the same catalytic residues with other esterase isozymes, their overall sequence identity is low. To examine evolutionary relationships, we constructed a minimum-likelihood phylogenetic tree and found that these proteins were placed into six different subclades within the same clan (CL0028; Finn et al., 2016). The BtsA homologs are restricted to *Moraxella* species and form a subclade distinct from other groups (Figure 8). Like BtsA, BioJ in *Francisella* species (Feng et al., 2014), BioK in Cyanobacteria (Rodionov et al., 2002) and BioV in *Helicobacter* species (Bi et al., 2016) are all restricted to a specific group of bacteria, whereas BioH and BioG are widely distributed among diverse bacteria (Shapiro et al., 2012). These data suggest that despite the proteins for the biotin precursor biosynthesis playing the same roles, each had followed its own evolutionary path. Thus, we posit that BtsA represents a new class of biotin synthesis carboxylesterase.

## Discussion

Our study revealed the biotin precursor synthetic pathway of *M. catarrhalis* and characterized the essential enzymes BioC and BtsA, that function sequentially in the same manner as the *E. coli* BioC-BioH classical pathway. BioC employs methylation, while BtsA acts demethylation to allow elongation of a temporarily disguised malonate moiety to a pimelate moiety by the fatty acid synthetic enzymes. The BioC-BioH pathway is the dominant, but not the only pathway for synthesis of the biotin pimeloyl moiety (Lin and Cronan, 2011). *B. subtilis* BioW (pimeloyl-CoA synthetase; Estrada et al., 2017; Manandhar and Cronan, 2017) and *Mesorhizobium loti* BioZ (Entcheva et al., 2002) were also reported to be involved in the biosynthesis of the pimeloyl moiety. Nevertheless, any BioW and BioZ homologs was not found in *M. catarrhalis* genome. Moreover, BtsA defines a new class of pimeloyl-ACP methyl ester esterase based on its low sequence identity with other isozymes and phylogenetic analyses. Thus, identification and characterization of BtsA supports the hypothesis that pimeloyl-ACP methyl ester esterase displays an unusual diversity among biotin-producing bacteria.

BioC was believed to an O-methyltransferase that methylated the free carboxyl of malonyl-ACP to initiate the biosynthesis of the pimeloyl moiety of biotin (Lin and Cronan, 2012). The similar result was observed in our study that the addition of malonyl-ACP into the reactions with the crude extracts prepared from the *E. coli* Δ*bioC* strain and McBioC can restore the growth of biotin auxotrophic strain ER90, demonstrating that malonyl-ACP is a potential substrate for McBioC. Furthermore, the biotin auxotroph of the Δ*btsA* strain was successfully rescued by expression of the *B. subtilis bioI* gene producing pimelate-ACP. Together with the esterase activity assays for BtsA function, we clearly demonstrated that BtsA recognized the ACP thioester of ω-methyl pimelic acid to give the product pimelate-ACP as the biotin precursor for biotin ring formation. This is different from the pimelate thioester linked to CoA in the BioW pathway, that converts pimelic acid to pimeloyl-CoA (Manandhar and Cronan, 2017).

Given the facts that most organisms require only miniscule amounts of biotin and the biotin synthetic pathway is metabolically expensive, tight regulation of biotin synthesis is expected. In *E. coli*, transcription of the biotin synthetic (*bio*) operon is directly regulated by BirA, the *E. coli* bifunctional repressor-biotin protein ligase (Barker and Campbell, 1980; Cronan, 1989). The two functions of BirA allow transcriptional regulation of the *bio* operon by DNA binding in respond to the intracellular concentrations of both biotin and biotin acceptor protein AccB (Cronan, 1988, 2014). The operator DNA binding requires an N-terminal winged helix-turn-helix domain which the *M. catarrhali*s biotin protein ligase (A4U55_08435) is lacking. Considering that *M. catarrhali*s lacks access to environmental biotin, it seems unable to regulate biotin synthesis in response to environmental biotin. It should be noted that in the *E. coli* genome the *bioH* gene is well-removed from the other biotin biosynthetic genes and is not regulated by the BirA repressor (Barker and Campbell, 1980). Like the “freestanding” BioH of *E. coli*, BtsA of *M. catarrhali*s is also encoded at a genome location far removed from its *bio* operon *bioAFCD* and *bioB* gene. Thus, whether *btsA* transcription is co-regulated with the other biotin synthesis genes remains to be solved.

Although either of the two proteins, A4U55_03600 and A4U55_05020 (BtsA) can functionally replace *E. coli* BioH, BtsA is the sole pimeloyl-ACP methyl ester esterase present in *M. catarrhalis*. Although A4U55_03600 shares essentially no sequence identity with BtsA or other pimeloyl-ACP methyl ester esterase isozymes, it should have a low promiscuous esterase. However, it is clearly designed for a distinct physiological role, but not for biotin biosynthesis. One possible reason is that A4U55_03600 would not perfectly interact with the ACP protein that carries the small molecule substrate, but BtsA does. Thus, resolving co-crystal structure of a complex of BtsA with pimeloyl-ACP methyl ester could represent a straight-forward way to gain a glimpse of this enigma. In addition, we investigated whether poor expression of A4U55_03600 explains its inability to support biotin precursor synthesis. This does not appear to be the case, because A4U55_03600 transcript level is comparable to *btsA* (data not shown).

Our data showed that deletion of *btsA* resulted into impaired abilities to invade respiratory epithelial cells and to survive within macrophages, indicating that biotin biosynthesis is required for *M. catarrhalis* virulence. Thus, our studies clearly conformed the role of biotin biosynthesis in bacterial virulence, that has been demonstrated in *M. tuberculosis* (Woong Park et al., 2011) and *F. tularensis* (Napier et al., 2012). In addition, understanding the mechanism of biotin synthesis in *M. catarrhali*s could provide targets for the development of antimicrobials to combat infection. BtsA is restricted to *Moraxella* species and thus BtsA inhibitor could be developed as promising antibacterial agents with specific activity against *Moraxella* cells. In conclusion, our data provide strong support for the future exploration of BtsA as a drug target, inhibitors of which should specifically target the *Moraxella* species but not otherwise affect the host microbiome.

## Data Availability Statement

The raw data supporting the conclusions of this article will be made available by the authors, without undue reservation, to any qualified researcher.

## Author Contributions

JJ and HB designed the experiments and wrote the manuscript. QZ and QY conducted the experiments and analyzed the data.

### Conflict of Interest

The authors declare that the research was conducted in the absence of any commercial or financial relationships that could be construed as a potential conflict of interest.
