# Supplementary material for: A Moraxella Virulence Factor Catalyzes an Essential Esterase Reaction of Biotin Biosynthesis
Source: Front Microbiol. 2020 Feb 11;11:148. doi: 10.3389/fmicb.2020.00148 (PMC7026016; doi:10.3389/fmicb.2020.00148)
Supplement: Supplementary file 1 [file Table_1.DOCX]

Supplementary Material

**Supplementary Table 1. *M. catarrhalis* biotin synthesis genes**

| Genes/genetic loci | Enzyme activity |
| --- | --- |
| *bplA* (A4U55_08435) | Biotin protein ligase |
| *bioC* (A4U55_00945) | SAM-dependent malonyl-ACP *O*-methyltransferase |
| *btsA* (A4U55_05020) | Pimeloyl-ACP methyl ester esterase |
| *bioF* (A4U55_00940) | 7-keto-8-amino pelargonic acid synthase |
| *bioA* (A4U55_00935) | 7,8-diaminopelargonic acid aminotransferase |
| *bioD* (A4U55_00950) | Dethiobiotin synthase |
| *bioB* (A4U55_04565) | Biotin synthase, SAM radical enzyme |

**Supplementary Table 2. Oligonucleotides used in this study**

| **Oligonucleotides** | **Sequence** |
| --- | --- |
| RS05165-F | AGCAGGAGGAATTCACAT**ATG**ACCCAAATTATCCTATCATC |
| RS05165-R | TCCGCCAAAACAGCCAAGCTTTTAAACTCTTAGATGACGCT |
| RS03745-F | AGCAGGAGGAATTCACAT**ATG**TCAAAATTAACTTCGGTAG |
| RS03745-R | TCCGCCAAAACAGCCAAGCTTTCAGCTTTTAATTAAGGCA |
| RS05165UP-EcoRI-F | ATAAGAATTC aagcacgccgacctgatg |
| RS05165UP-KpnI-R | ATATGGTACC TGGTGATGTTGGCTATAC |
| RS05165Dn-ApaI-F | ATATGGGCCC tcggtcatcatgtatttc |
| RS06165Dn-SacI-R | TTAGGAGCTC GATGGCATGAGTATCACA |
| BioCUP-EcoRI-F | CAGTGAATTC CGTCATCAATCAAGTTGAT |
| BioCUP-KpnI-R | ACATGGTACC GATTTGATGATAAAGCGGA |
| BioCDn-ApaI-F | ATATGGGCCC ATGTTGGAGCAAGCAAAG |
| BioCDn-SacI-R | ATCAGAGCTC ACTATCTCATAAGCCTCTG |
| 184-Km-F | TTATGCCTCTTCCGACCATC |
| 184-Km-R | ACTGAATCCGGTGAGAATGG |
| McUP-KpnI-L | AATTGGTACCgtgttctgtcttagttgca |
| McUP-ApaI-R | ATCTAGGGCCC ACACAAGTTGTTCTTTAGT |
| McDn-XbaI-L | GCGGCTCTAGAttgtcaatacaagatactt |
| McDn-SacI-R | ATATGAGCTCAGATCGTCGCCTTGGTAG |
| McBtsA-XhoI-F | ATATCTCGAGttggcaaaggcagagcgt |
| McBtsA-SalI-R | GCCTGTCGACTTAAACTCTTAGATGACGCT |
| McBioC-XhoI-F | ATATCTCGAG TTGGCAGCGATTAGCCTT |
| McBioC-2 | AAAGCGGATTTTGTCATCGCCTAAATCTTAGTCAA |
| McBioC-3 | TTGACTAAGATTTAGGCGATGACAAAATCCGCTTT |
| McBioC-SalI-R | GACCGTCGAC TTATAAAGGCGAACTCAT |
| BsBioI-XhoI-F | ATATCTCGAG TATGGATGGCAAGGTTGC |
| BsBioI-2 | TGACGATGCAATTGTCATCGCCTAAATCTTAGTCAA |
| BsBioI-3 | TTGACTAAGATTTAGGCGATGACAATTGCATCGTCA |
| BsBioI-SalI-R | GACTGTCGAC TTATTCAAAAGTCACCGGCA |
| CM-F | CTGACTGCAGATCCGCCATATTGTGTTGA |
| CM-R | ATATGGATCCCGCAGAACTGGTAGGTATGGA |
| RS05165-S117A-F | CCAGTTTCCCATTATATCATGGGGCATGCGATGGGGTCATTCATTGTGCGAACGG |
| RS05165-S117A-R | CCGTTCGCACAATGACCCCATCGCATGCCCCATGATATAATGGGAAACTGG |
| RS05165-D254A-F | ATTTTATTAATCAGTGGCAATAATGCGCCCGTAGGCAATATGGGTCAAGACA |
| RS05165-D254A-R | TGTCTTGACCCATATTGCCTACGGGCGCATTATTGCCACTGATTAATAAAAT |
| RS05165-H287A-F | GTGTTCAACTTTATCCTAATATGCGTGCGGAGCCACTGCATGAAAAACAAGCACA |
| RS05165-H287A-R | TGTGCTTGTTTTTCATGCAGTGGCTCCGCACGCATATTAGGATAAAGTTGAACAC |

**
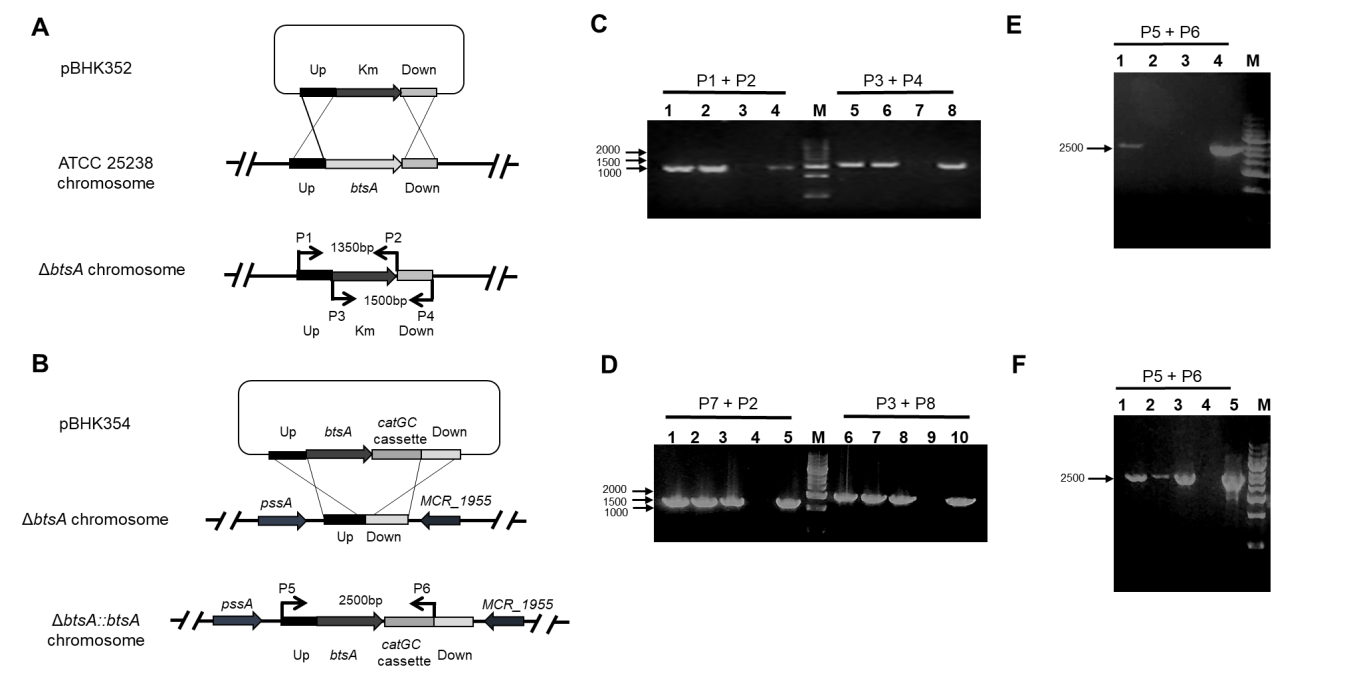
**

**Figure S1. Construction and PCR confirmation of the Δ*btsA*,** Δ***bioC,* Δ*btsA::btsA* and Δ*bioC::bioC* strains. (A)** Illustration of the construction of the Δ*btsA* strain using pCM184 system by double cross recombination. The successful knocked out mutants were confirmed by primer pair P1/P2 and P3/P4. **(B)** Illustration of the construction of the complemented strain Δ*btsA::btsA* using chromosomal complementation system by natural transformation via allelic exchange. **(C)** PCR confirmation of Δ*btsA* strain. The expected product size of Δ*btsA* using primer set P1/P2 and P3/P4 is 1350 bp and 1500 bp, respectively. From left to right, the PCR templates (Lane 1-4 and Lane 5-8) are the genomic DNAs of the Δ*btsA* strain colony 1, colony 2, *M. catarrhalis* ATCC 25238, plasmid DNA of pBHK352, respectively. **(D)** PCR confirmation of the Δ*bioC* strain. The Δ*bioC* strain was obtained by the same genetic manipulation as construction of the Δ*btsA* strain shown in (A). The expected product size of Δ*bioC* using primer set P6/P2 and P3/P7 is 1350 bp and 1500 bp, respectively. From left to right, the PCR templates (Lane 1-5 and Lane 6-10) are the genomic DNAs of the Δ*bioC* strain colony 1, colony 2, colony 3, *M. catarrhalis* ATCC 25238 and the plasmid DNA of pBHK353, respectively. **(E)** PCR confirmation of the Δ*btsA::btsA* strain. The expected size of amplified PCR product using primer pair P1/P5 is about 2.5 kb. The PCR templates from Lane 1-4 are the genomic DNAs of the complemented Δ*btsA::btsA* strain, no template (negative control), *M. catarrhalis* ATCC 25238, plasmid DNA of pBHK354, respectively. **(F)** PCR confirmation of the Δ*bioC::bioC* complemented strain. The expected size of amplified PCR product using primer pair P8/P5 is about 2.5 kb. The PCR templates from Lane 1-5 are the genomic DNAs of the Δ*bioC* strain colony 1, colony 2, colony 3, no template (negative control), plasmid DNA of pBHK355, respectively. The molecular weight of DNA marker (M) is presented in base pair (bp). The primers P1-P8 are presented in Supplementary Table 2 as followed: P1, RS05165UP-EcoRI-F; P2, 184-Km-R; P3, 184-Km-F; P4, RS05165Dn-SacI-R; P5, McUP-KpnI-L; P6, CM-R; P7, BioCUp-EcoRI-F; P8, BioCDn-SacI-R.


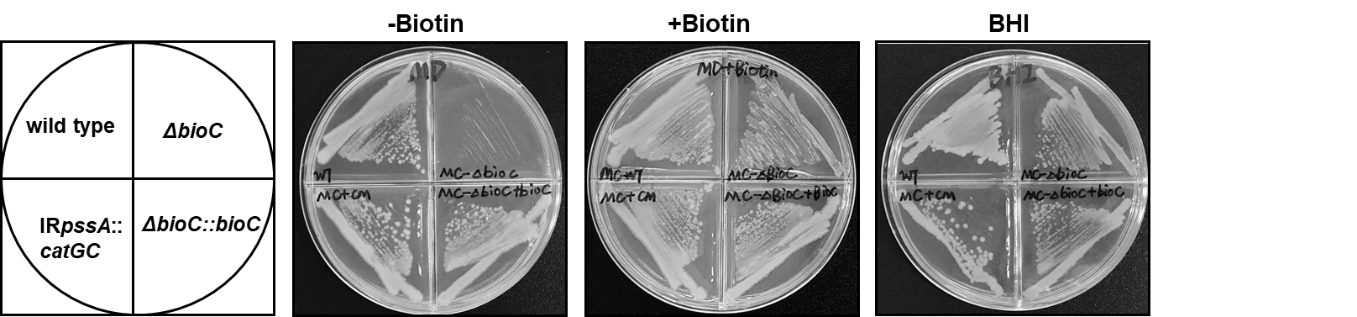


**Figure S2. *M. catarrhalis* requires *bioC* for growth.** Growth phenotypes of various *M. catarrhalis* strains were observed at 37 ^0^C on the defined medium with or without biotin (10 nM) supplementation. The plates were incubated at 37°C for 2 days. Growth on BHI plates was used as a positive control. The tested strains are wild type (upper left sector), Δ*bioC* (ATCC 25238), IR*pssA*::*catGC* (BHKS368), Δ*bioC::bioC* (BHKS376).

**
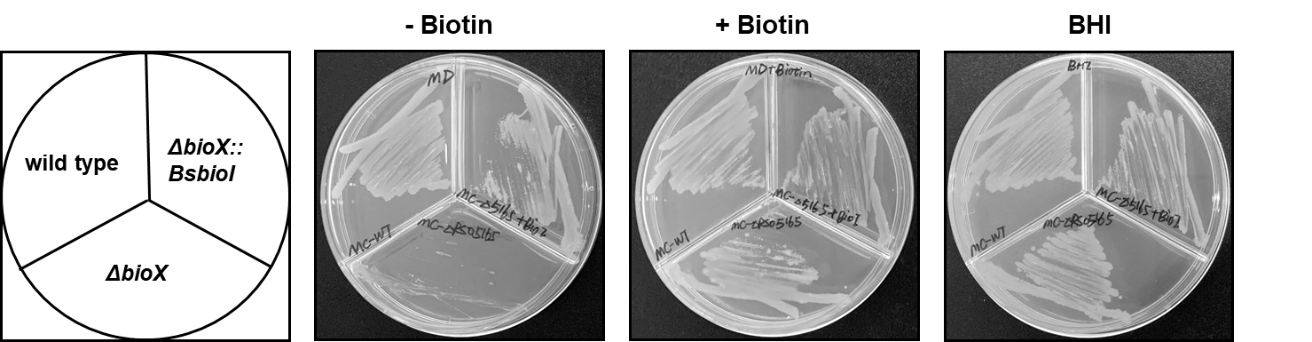
**

**Figure S3. Expression of *B. subtilis bioI* restored the growth of the Δ*btsA* strain on biotin-free minimal medium.** Transformants of strain were grown at 37 ^0^C on defined minimum medium (with or without biotin) and nutritious medium BHI. The strains tested were: wild type (ATCC 25238), Δ*btsA*::*BsbioI* (BHKS449) and Δ*btsA* (BHKS211). *B. subtilis bioI* allowed growth of the *M. catarrhalis* Δ*btsA* strain without biotin supplementation.


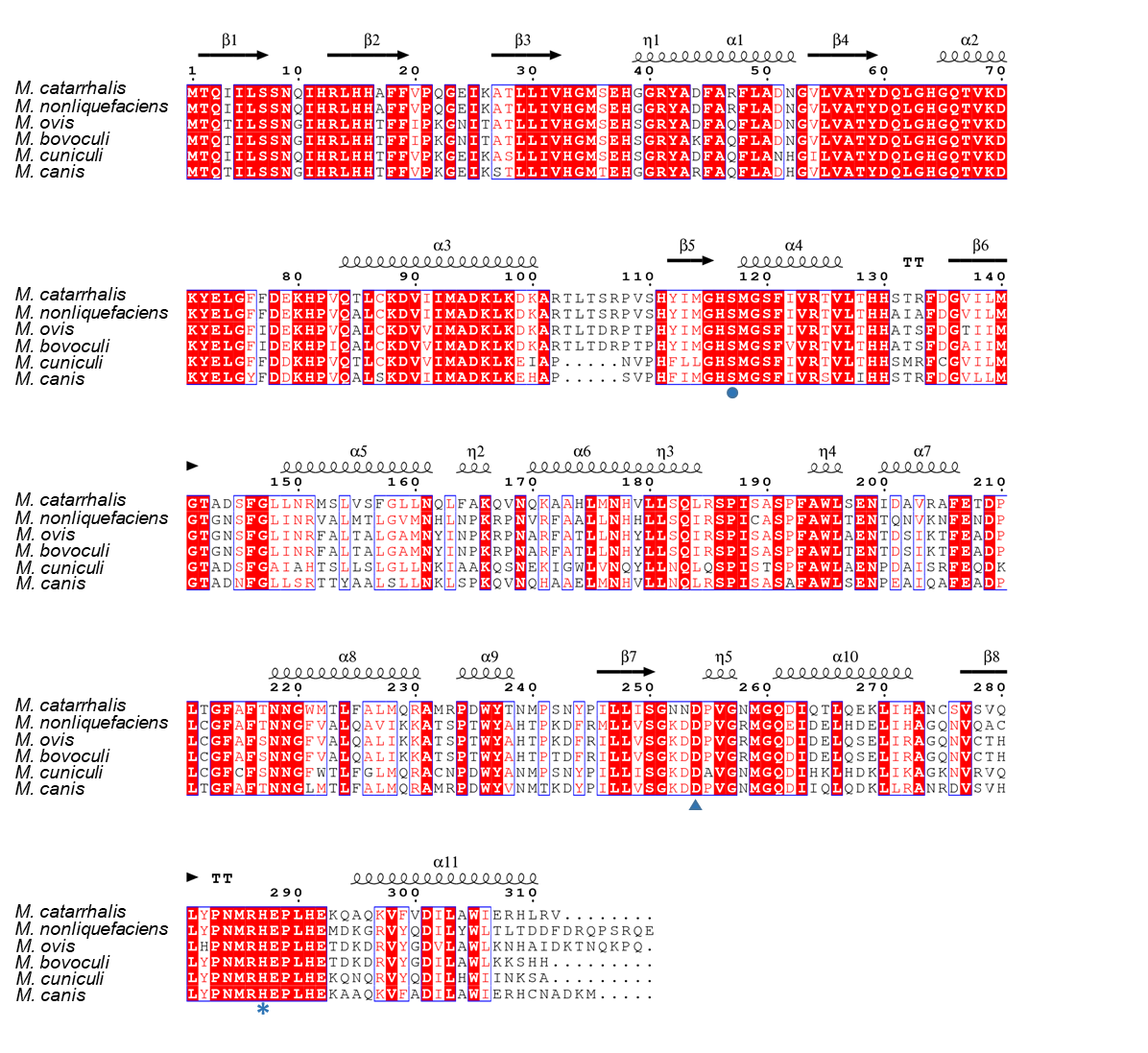


**Figure S4. Multiple protein sequence alignments of six BtsA homologues from different *Moraxella* species.**

The predicted protein sequences of the BtsA homologues are from *M. catarrhalis* (Accession No. WP_003668336), *M. canis* (Accession No. WP_049236735), *M. ovis* (Accession No.), *M. nonliquefaciens* (Accession No. WP_066892133), *M. bovoculi* (Accession No. WP_046697870), and *M. cuniculi* (Accession No. WP_076556080). The identical residues are in white letters with red background, and the varied residues are in black letters. The predicted protein secondary structure of BtsA is given in cartoon form, which is based on CPHmodels 3.0 Server. Designations: α: alpha-helix; β: beta-sheet; T: Turn; η: coil. The solid circle, triangle and asterisk denote the active-site residues.


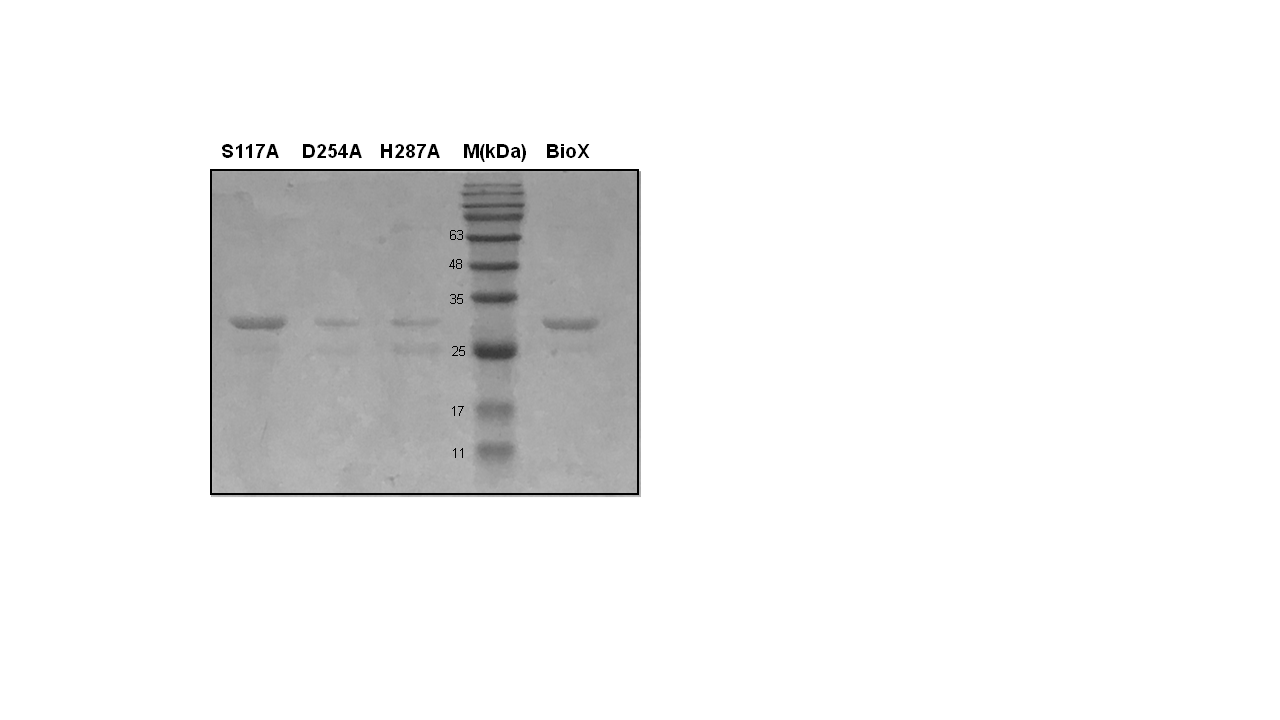


**Figure S5. SDS-PAGE analysis of the purified BtsA and three mutant BtsA proteins.** Protein samples were separated by electrophoresis using 4-20% gradient SDS-PAGE. The molecular weight of BtsA and its mutants (S117A, D254A, H287A) is about 30 kDa.


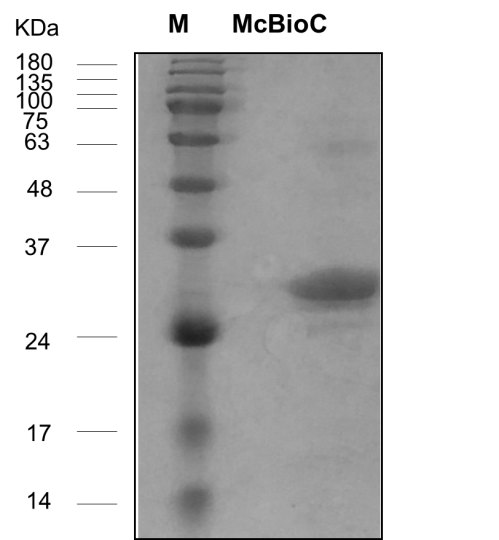


**Figure S6. SDS-PAGE analysis of the purified McBioC.** Protein samples were separated by electrophoresis using SDS-PAGE. The molecular weight of BioC is about 33 kDa.


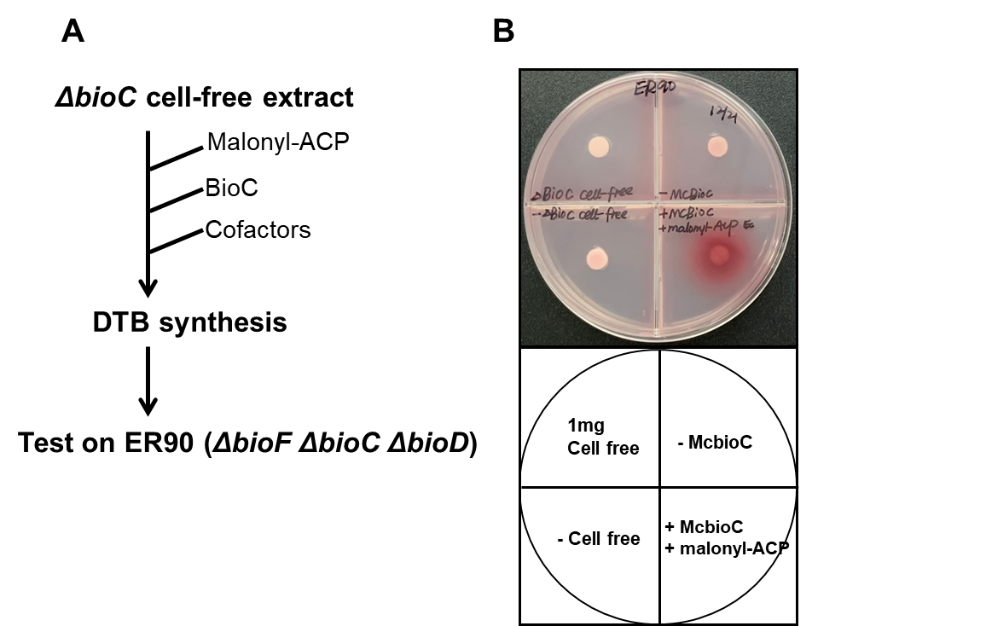


**Figure S7. Bioassay of McBioC function in the overall biotin synthetic pathway. (A)** Scheme of the *in vitro* DTB synthesis system of BioC. **(B)** Restoration of DTB synthesis to the *∆bioC* extract by addition of malonyl-ACP methyl ester and BioC. The upper left quadrant contained the *E. coli* Δ*bioC* cell free extract (1 mg) only. The upper right quadrant contained all components required for DTB synthesis except BioC whereas the samples spotted on the lower left quadrant lacked the *E. coli ∆bioC* strain cell free extract. The lower right quadrant contained all components required for DTB synthesis including McBioC and its substrate malonyl-ACP.


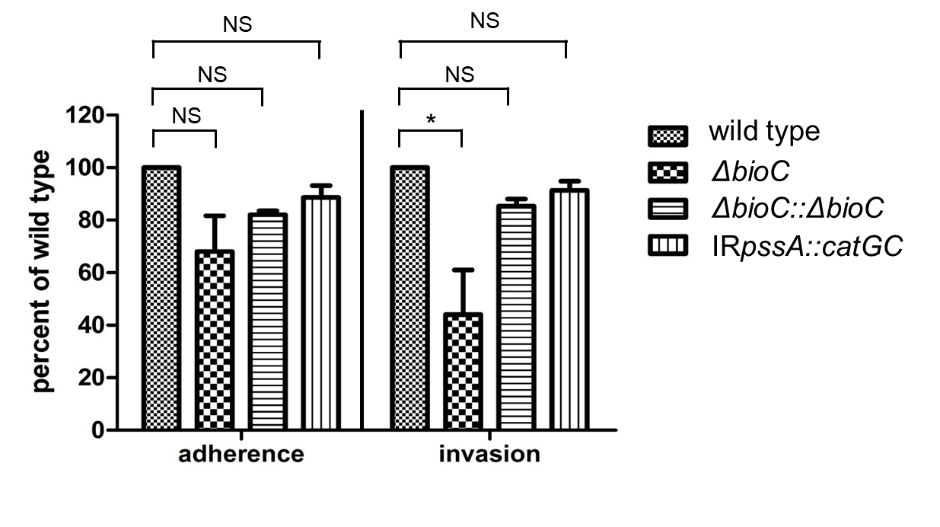


**Figure S8. The BioC is required for the *M. catarrhalis* invasion to human respiratory epithelial A549 cells.** The cells were infected and, after 30 min for adherence and 3 h for invasion, total cell-associated bacteria or intracellular bacteria, respectively, were quantitated by dilution plating. The y axis shows results relative to those for the wild type. Data are represented as means ± SD of three independent experiments. *P < 0.05.
